# Nurse educators' experiences and perceptions using generative artificial intelligence: a systematic review

**DOI:** 10.1186/s12909-026-10113-0

**Published:** 2026-08-15

**Authors:** Ani Henttonen, Maria Christidis, Helena Kullenberg, Taina Sormunen, Margareta Westerbotn, Ann Hägg-Martinell

**Affiliations:** 1 Department of Health Promoting Science, Sophiahemmet University, Stockholm, Sweden; 2 Department of Nursing Science, Sophiahemmet University, Stockholm, Sweden; 3 Department of Neurobiology, Care Sciences and Society, Karolinska Institutet, Stockholm, Sweden

**Keywords:** Competency development, Generative artificial intelligence, Nurse education, Nurse Educators

## Abstract

**Background:**

The rapid uptake of generative artificial intelligence (GenAI) in higher education has increased both enthusiasm and concern. While students’ use of GenAI has been widely discussed, empirical research focusing on nurse educators’ own experiences and perceptions remains limited. This systematic review synthesizes evidence on nurse educators’ experiences of using generative artificial intelligence in teaching.

**Methods:**

A systematic literature review was conducted in accordance with PRISMA 2020 guidelines. Searches were performed in PubMed, CINAHL, Web of Science, and ERIC. Peer-reviewed empirical studies published in English were included. Two reviewers independently screened records, extracted data, and conducted quality appraisal using established tools. Due to methodological heterogeneity, results were synthesized thematically.

**Results:**

Thirteen studies were included, representing a total of 3082 participants. Two overarching themes were identified: (1) Nurse educators’ opportunities and challenges using Generative AI in teaching, and (2) Nurse educators’ competence and ways of using Generative AI. Educators described Generative AI as a potentially valuable resource for teaching efficiency and organizational and pedagogical inspiration. They expressed concerns relating to their loss of professional roles, academic integrity, and erosion of critical thinking related to students. Experience with Generative AI, institutional position, organizational policy and support influenced educators’ attitudes, confidence, and use.

**Discussion:**

The findings reveal a tension between optimism about Generative AI’s pedagogical potential and apprehension about its ethical, educational, and professional implications. Educators’ calls for clearer policies, competency development, and institutional support highlight the need for systematic capacity-building.

**Conclusion:**

Generative AI's value depends on educators' skills, supportive policies, and intentional use, making structured training and governance essential for integration in nurse education.

**Supplementary Information:**

The online version contains supplementary material available at 10.1186/s12909-026-10113-0.

## Background

Artificial intelligence (AI) is an umbrella concept encompassing computational systems that enable machines to perform cognitive tasks traditionally associated with human intelligence, including learning, reasoning, and decision making [1]. A subset of these technologies is generative AI (GenAI), which refers to advanced systems that are trained through prompts in datasets to produce new content, based on the data patterns [2]. The production can include text, language, and/or images. GenAI comes in the form of open, standalone tools, such as ChatGPT, and as AI integrated into applications and platforms, for example Microsoft Office Copilot (Microsoft, [3]. GenAI entered mainstream public use in 2022 and has since rapidly permeated society, particularly the education sector. As these technologies become embedded in academic practices, there is a growing need for research that examines nurse educators’ perspectives, as well as the deeper systemic implications of these technologies [4].

In this review, nurse educators and nursing faculty (hereafter referred to collectively as nurse educators) are defined as academics involved in teaching and facilitating learning within nurse education programmes in higher education institutions (HEI). While many nurse educators are registered nurses, nursing programmes may also employ educators from other health health professions. Consequently, the professional backgrounds, roles and qualification requirements of nurse educators can vary across institutions and countries, both within Europe and globally.

AI policies and guidelines in HEIs offer guidance for students and educators but require further development to address their integration, and practical use in teaching and learning. In a study by Erhardt et al. [5], the primary focus of policies and guidelines in Swedish HEIs concerned good academic practice, including transparency, responsibility, and challenges with misconduct. However, the study also highlighted aspects of GenAI that required further attention, including its use and governance in education, information governance (such as sensitive data, and copyright), ethical and social implications (such as bias and equitable access), and the need to strengthen nurse educators’ competence in using GenAI. GenAI technologies are rapidly evolving, which requires institutions to sustain flexibility and remain agile, by continuously updating pedagogical strategies and support systems to ensure responsible and effective integration [6–8].

This dynamic is equally evident in the field of nurse education, where the diverse and continuously evolving models of GenAI were shown to exhibit inconsistencies due to linguistic diversity, which in turn may undermine reliability and reduces the accountability of their output [9]. It is therefore imperative to keep pace with the rapid advancement of GenAI technologies.

According to the World Health Organization (WHO) [10] nurse teacher core competency framework, nurse educators are expected to draw on pedagogical knowledge to guide the governance, organization, and analysis of adult learning. GenAI can be viewed as a tool for nurse educators’ work, for instance, in the design of case-based scenarios, provision of formative feedback, and the development of clinical multiprofessional learning activities in health professions education.

Research showed that education delivered by an interactive chatbot supported independent nursing care management and decision-making skills among nursing students [11]. Moreover, engaging with AI in a critical manner may encourage students to reflect on how these technologies shape their knowledge and decision-making in health care contexts [12]. However, concerns have also been raised among students regarding the ethical use of GenAI, particularly the risk that relying on such tools for learning may lead to diminished competence in foundational clinical judgement skills [13].

For students in the health professions, GenAI technologies can support conscientious learning as auxiliary tools, while professional responsibility, ethical accountability, and judgement remain core human competencies [14].

While initial scholarly discourse has focused on the ethical aspects of GenAI integration in nurse education, there remains a paucity of research concerning its practical application by nursing educators in their teaching roles. This systematic review sets out to review recent literature on nurse educators' experiences and perceptions regarding use of GenAI in teaching.

## Methods

### Design

A systematic literature review was conducted to summarize selected research on a specific question using a structured, transparent, and reproducible method [15]. A systematic review adheres to a defined research question and employs structured methods to minimize bias and to increase the reliability of its conclusions [16].

This systematic review was conducted and reported in accordance with the Preferred Reporting Items for Systematic Reviews and Meta-Analyses (PRISMA) 2020 statement, which primarily provides guidance for the reporting of systematic reviews describing and evaluating practices and interventions [17].

### Data inclusion

To ensure a systematic and targeted literature search, eligibility criteria were defined a priori using the Population, Intervention/Exposure, Comparator, and Outcome (PICO/PECO) framework where applicable [18]. Studies were included if they (1) addressed the predefined population, (2) were original empirical studies, (3) were published in peer-reviewed journals, and (4) were written in English. Articles that focused on other populations or evaluated GenAI-software were excluded. Reviews, editorials, conference abstracts, and non-peer-reviewed publications were also excluded (Table 1).

**Table 1:** Inclusion and exclusion criteria

Inclusion criteria	Exclusion criteria
Nurse educators, teachers and faculty	Students
Original research	Articles evaluating AI-softwares
Articles published in English	Reviews of literature
Publications from 2022 to October 2025	Editorials
Peer reviewed articles	Conference abstracts

### Search strategy

To optimize the sensitivity and precision of the database searches and ensure alignment with the review aim, a professional academic librarian with expertise in systematic search was consulted during the development of the search strategy. Search blocks and search terms were constructed to operationalise the selection criteria and to capture the key concepts of the review. The search blocks were based on the following terms:

“generative artificial intelligence” AND “nurs* educat*” OR “nurs* facult*”

For the purposes of this systematic review, Generative Artificial Intelligence was operationally defined as artificial intelligence systems capable of generating novel content based on learned patterns from training data. Given the rapidly evolving soft-ware terminology in the field, the search strategy involved both generic descriptors, (eg., generative AI, GenAI), and names of specific GenAI applications, (e.g., ChatGPT, Gemini, Perplexity). To maximize sensitivity, broader AI-related terms, (machine learning, machine intelligence) were also included [19]. The search was limited to publications from 2022 onwards, as 2022 marked the beginning of the widespread public availability and adoption of GenAI technologies.

The electronic databases used were PubMed from inception, Web of Science, Education Resources Information Center (ERIC) (via the platform of ProQuest), and Cumulative Index to Nursing and Allied Health Literature CINAHL (via the platform of EBSCO host). PubMed and CINAHL were selected as the primary databases for identifying international research literature in health professions education. Web of Science and ERIC were used for publications with the focus on academic educational research. Comprehensive literature search in the databases Cinahl, PubMed and Web of Science were conducted between March and October 2025, with the final search (ERIC) completed October 2025. Accordingly, eligible publications dated from Januari 2022 to 8 October 2025.

**Fig. 1:**
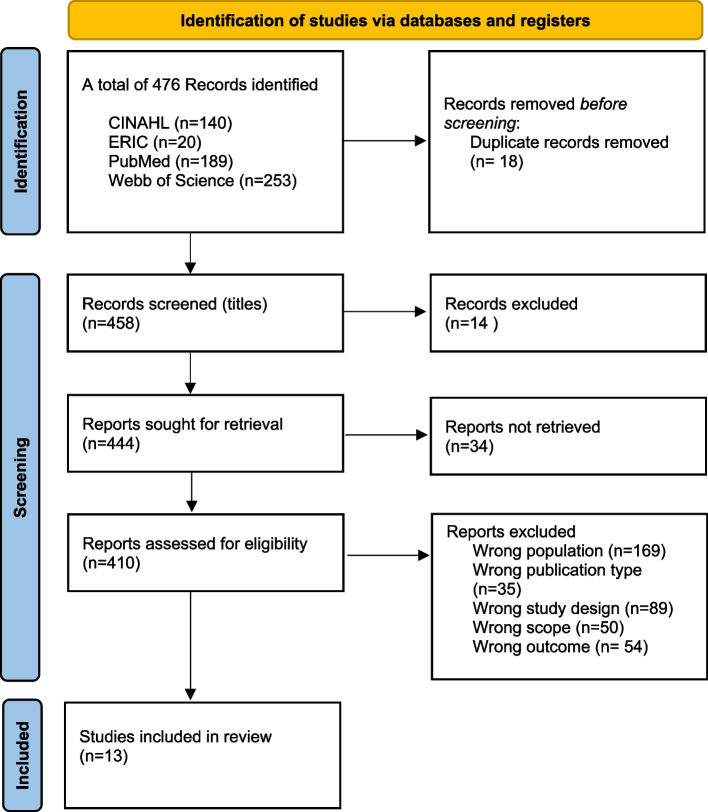
PRISMA Flow diagram on identification, screening, and inclusion of the samples of articles

The process of identification, screening, and inclusion is illustrated in the PRISMA Flow diagram (Fig. 1).

### Study selection

All records identified through the database searches were imported into the web-based screening tool Rayyan [20]. Following the removal of duplicates, two reviewers (AH, HK) independently screened titles and abstracts against the eligibility criteria including publication type, population, study design and scope. Full-text articles were retrieved for all potentially relevant studies and assessed independently by the same reviewers.

During title, abstract and full-text screening, studies were assessed against the operational definition of GenAI to ensure conceptual alignment with the review objective, that is, inclusion was restricted to studies reporting nurse educators’ experiences and perceptions of GenAI rather than analytical or non-generative AI technologies [21]. Studies examining nurse educators’ GenAI readiness, competence, self-efficacy or attitudes were eligible when these constructs reflected or informed educators’ experiences, use or perceptions of GenAI in nurse education.

Any disagreements regarding study eligibility were resolved through discussion between the reviewers, with a third reviewer (MC) consulted when consensus could not be reached. Following the screening process, thirteen studies met the eligibility criteria and were included in the review.

### Risk of bias assessment

Two reviewers (MW and AHM) independently evaluated the methodological quality of the included studies using the JBI Critical Appraisal Checklist for Analytical Cross-Sectional Studies [22], the JBI Critical Appraisal Checklist for Qualitative Research [23] and the Mixed Methods Appraisal Tool (MMAT) [19, 24], as appropriate to the study design. Any discrepancies in the quality assessments were discussed and resolved through consensus, with a third reviewer (MC) consulted when agreement could not be reached. The results of the quality appraisal were used to describe the methodological strengths and limitations of the included studies; no studies were excluded based on quality assessment.

### Data extraction

Data were extracted independently by two reviewers (AH, HK). Extracted information included study characteristics, i.e., author, year, country, study design, population characteristics, interventions or exposures, outcomes, and key findings. To ensure accuracy in monitoring, the extraction process included condensation (abstraction) of data, as described by [15]. Any discrepancies in the extracted data were resolved through discussion between the reviewers, with a third reviewer (AHM) consulted when consensus could not be reached.

### Data analysis

Due to methodological heterogeneity across the included studies, a thematic analysis according to Braun and Clarke’s [25] approach was undertaken. All authors were involved throughout the analytic process, with analytical decisions being discussed and refined during monthly research group meetings held either online or in person. First, the included studies were read repeatedly to facilitate familiarisation with the data and to identify evidence relevant to the review aim. Two authors, (AH and HK) independently coded data extracts relevant to the review aim, generating initial codes that captured recurring patterns related to nurse educators’ experiences and perspectives of GenAI. The coded data were subsequently compared and discussed among all authors. Any discrepancies in coding and interpretation were resolved through repeated discussions among all authors and by revisiting the original study findings until consensus was reached [25]. Codes representing similar patterns across the dataset were grouped together and organised into candidate themes. Theme development involved an iterative process of moving between the coded data, candidate themes, and the original study findings. All authors contributed to reviewing, refining and defining the themes to ensure coherence, internal consistency, and alignment with the underlying data. Through this collaborative process, candidate themes were refined, merged, separated or redefined where necessary to reduce conceptual overlap and enhance clarity. The final thematic structure was developed through repeated discussion and comparison of the coded data and candidate themes, resulting in two overarching themes*: Nurse educators’ opportunities and challenges using GenAI in teaching,* and *Nurse educators’ competence and ways of using GenAI.*

## Results

In this section, we present two sets of results. First, we describe the characteristics of the included studies in a table form, including their study designs, participant numbers, data collection methods, aims, key findings and associated themes. Second, we present the results of the thematic analysis.

The 13 selected studies examining nursing educators’ experiences and perceptions of GenAI in teaching represent research conducted across ten countries.

These include Australia (*n* = 4), Bangladesh (*n* = 1), Egypt (*n* = 4), India (*n* = 1), Jordan (*n* = 1), Philippines (*n* = 1), Saudi Arabia (*n* = 2), South Korea (*n* = 2), Turkey (*n* = 1), and USA (*n* = 3) comprising a total sample of 3082 participants. Two of the publications also involved cross-national comparisons, analyzing data from two and four countries, thereby describing cultural and practical aspects in relation to GenAI as a comparative variable within their study designs [26, 27]. The included studies represented six quantitative, five qualitative and two mixed-methods research designs, and they were published between 2024 (*n* = 1) and 2025 (*n* = 12).

Table 2 provides an overview of the articles included.

**Table 2:** Overview of included research articles

Authors, year, country	Design, participants, data collection, analysis	Aim	Key findings	Associated theme(s)
Almalki et al. [26] Saudi Arabia, Philippine, India, Egypt	Design: Cross-sectional study Sample: 1021 nurse educators Data collection: Online survey. Analysis: ANOVA	To determine the impact of the integration of artificial intelligence in nursing education, specifically examining nurse educators’ perceptions of benefits, risks, trust, exposure, and the influence of culture in a cross-national context	Consistent perceived benefits across settings; context-specific implementation strategies; increased familiarity fostered trust	***Nurse educators’ opportunities and challenges using Generative AI in teaching*** ***Nurse educators’ competence and ways of using Generative AI***
Asal et al.[28] Egypt	Design: Cross-sectional study Sample: 600 nurse educators Data collection: Self-report scales on digital pedagogy competence, pedagogical innovation, and AI readiness Analysis: Inferential statistics	To explore the perspectives of nursing faculty members regarding the integration of ChatGPT into nursing education	Digital competence and AI readiness associated with pedagogical innovation; digital skills facilitated AI adoption; training needs highlighted	***Nurse educators’ opportunities and challenges using Generative AI in teaching*** ***Nurse educators’ competence and ways of using Generative AI***
Durmuş Sarıkahya et al. [29] Turkey	Design: Qualitative Sample: 14 nurse educators Data collection: semi-structured interviews Analysis: reflexive thematic analysis	To explore the perspectives of nursing faculty members regarding the integration of ChatGPT into nursing education	Benefits: time-saving, language refinement and teaching support. Challenges: missing citations, superficial content, limited relevance to person-centred care, information overload, reduced critical thinking, lack of practical skill development and misuse monitoring	***Nurse educators’ opportunities and challenges using Generative AI in teaching***
Ehmke et al., [30] USA	Design: Cross- sectional study Sample: 72 nurse educators Data collection: Online survey Analysis: Descriptive statistics	To investigate nursing faculty’s knowledge, skills, and attitudes toward integrating Artificial Intelligence (AI) into education, examining differences by degree type and the influence of policies or syllabi on AI integration	AI policy engagement and educational level influenced GenAI readiness; higher qualifications associated with greater AI competence, lower qualifications with more positive attitudes	***Nurse educators’ opportunities and challenges using Generative AI in teaching*** ***Nurse educators’ competence and ways of using Generative AI***
El-Sayed et al. [31] Egypt, Saudi Arabia	Design: Cross -sectional Sample: 570 nurse educators Data collection: Self-reported online questionnaire assessing creativity-nurturing behaviours, AI competence self-efficacy, and implementation climate Analysis: Descriptive and inferential statistics	To examine the relationship between evidence-based practice climate and creativity-nurturing behaviors among nurse educators, with a specific focus on the moderating role of AI competence self-efficacy	Evidence-based practice climate and AI competence self-efficacy predicted creativity-supporting behaviours; higher AI confidence strengthened this association	***Nurse educators’ opportunities and challenges using Generative AI in teaching***
Eltaybani et al., [32] Egypt	Design: Mixed Method Sample: 56 nurse educators Data collection: Questionnaire and interviews Analysis: Descriptive statistics and qualitative content analysis	To explore how nurse educators and students use Large Language Models (LLMs) for academic purposes and their perceived challenges	Traditional approaches and student autonomy discouraged use, whereas efficiency gains supported applications in writing, assessment and clinical teaching. Challenges included inaccuracies, limited functionality and unfamiliarity with LLMs	***Nurse educators’ opportunities and challenges using Generative AI in teaching***
Hashish et al., [33] Saudi Arabia	Design: Mixed-methods/Convergent approach Sample: 40 + 15 nurse educators Data collection: Self-reported questionnaire and semi-structured interviews Analysis: Descriptive and inferential statistics, thematic analysis	To explore nursing students’ and educators’ perspectives on using ChatGPT in academia through a mixed-methods approach	Prior GenAI exposure associated with greater knowledge and more positive attitudes; older educators reported lower knowledge and higher concern. Uses included instructional design, academic writing and content generation	***Nurse educators’ opportunities and challenges using Generative AI in teaching*** ***Nurse educators competence and ways of using Generative AI***
Hong et al. [34] South Korea	Design: Cross-sectional Sample: 120 nurse educators Data collection: Online questionnaire. Analysis: Descriptive statistics	To explore the perceptions and experiences of nurse educators in South Korea regarding the use of generative artificial intelligence	Positive perceptions despite limited experience; AI supported educator competence, student learning and teaching confidence. Ethical and legal concerns, curricular uncertainty and scepticism towards replacing nurse educators remained	***Nurse educators’ opportunities and challenges using Generative AI in teaching*** ***Nurse educators’ competence and ways of using Generative AI***
Kim et al.[35] South Korea	Design: Descriptive qualitative Sample: 17 nurse educators Data collection: Individual interviews Analysis: Thematic analysis	To explore Korean nurse educators’ perceptions of GAI in nursing education and identified the steps necessary for its successful integration	Limited familiarity despite interest in educational use. Perceived benefits included information retrieval and lesson design; concerns centred on workload, accuracy, ethical/legal risks and threats to the educator role. Need for training, guidance and institutional support identified	***Nurse educators’ opportunities and challenges using Generative AI in teaching*** ***Nurse educators competence and ways of using Generative AI***
Lane et al., [36] USA	Design: Case-based descriptive qualitative design on written Sample: 95 nurse educators Data collection: electronic and physical written responses Analysis: Thematic analysis	To understand the perspectives on AI among informed audience of nurse educators	GenAI was perceived as both time-saving and time-consuming, while simultaneously facilitating and impeding innovation, critical thinking and routine academic tasks	***Nurseeducators’ opportunities and challenges using Generative AI in teaching*** ***Nurse educators’ competence and ways of using Generative AI***
Rony et al. [37] Bangladesh	Design: Qualitative, phenomenological Sample: 14 nurse educators Data collection: individual semi-structured interviews and focus group discussions Analysis: Thematic analysis	To explore nursing educators’ perspectives on integrating AI into academic settings	Improved teaching efficiency and personalised learning; barriers included limited training, support and infrastructure. Concerns involved data privacy and algorithmic bias; readiness varied across professional roles	***Nurse educators’ opportunities and challenges using Generative AI in teaching***
Saleh et al. [27] Jordan, USA	Design: Cross-sectional Sample: 474 nursing faculty Data collection: Self-reported online questionnaire Analysis: Descriptive statistics and MANCOVA	To examine faculty perceptions of artificial intelligence (AI) chatbots in nursing education, focusing on their usage patterns, perceived benefits, and limitations	Benefits recognised, but concerns included job insecurity, reduced faculty motivation, implementation challenges, misinformation, limited adaptability and the perceived replacement of faculty roles	***Nurse educators’ opportunities and challenges using Generative AI in teaching*** ***Nurse educators’ competence and ways of using Generative AI***
Summers & Lee [38] Australia	Design: Descriptive qualitative Sample: 12 nursing faculty members Data collection: Interviews Analysis: Thematic analysis	To explore: What are the views of nursing faculty about the use of GenAI tools in nursing student assessments? When a GenAI tool has possibly been used in an assessment, how do they grade that assessment? What are the characteristics they have noticed about GenAI in student assessments?	Potential to support structured learning; concerns centred on equity, assessment validity and reduced critical thinking. Clear policies, detection tools and ethical guidance were recommended	***Nurse educators’ opportunities and challenges using Generative AI in teaching*** ***Nurse educators’ competence and ways of using Generative AI***

### Theme 1: Nurse educators’ opportunities and challenges using Generative AI in teaching

Included studies described nurse educators’ perceptions of the opportunities and challenges associated with integrating GenAI into teaching. Several studies explored factors associated with these perceptions, including educators’ age [27] cultural context [26], academic role [35] and readiness for pedagogical innovation [28]. Age-related differences were reported, with nurse educators aged 50 years and older demonstrating lower levels of GenAI knowledge than their younger colleagues [33]. However, the factors most consistently associated with educators’ perceptions were prior exposure to GenAI and practical experience of its use [26, 28, 33, 35]

Nurse educators generally perceived GenAI as a valuable resource for academic and teaching-related activities [29, 32]. Educators described GenAI as supporting teaching efficiency [37], reducing time spent on routine tasks [36], facilitating information synthesis [32] and providing inspiration for teaching patient education and written care plans [29]. Educators also reported that GenAI could support professional tasks through improved efficiency, easier access to information, and assistance with academic work.

At the same time, several studies highlighted the coexistence of positive and negative perceptions of GenAI among nurse educators [27, 36, 38]. Educators frequently rated the perceived benefits and concerns associated with GenAI at similar levels, suggesting a balanced but ambivalent view of its use [27]. Educators described GenAI as simultaneously time-saving and time-consuming, capable of both fostering and constraining innovation, and as both supporting and potentially undermining critical thinking [36, 38]. Some educators viewed GenAI as a useful aid for routine responsibilities, whereas others regarded it as a distraction that could increase workload rather than reduce it [38].

Experience with GenAI appeared to shape these perceptions. In a cross-national study, [26] found that repeated hands-on use of GenAI in professional contexts was an important factor influencing educators’ perceptions of both its benefits and risks. Similarly, [35] reported that educators’ experience of using GenAI within their educational roles was associated with more positive attitudes towards the technology. Exposure to GenAI was identified as the strongest predictor of trust and perceived benefits in a study involving educators from India, Egypt, the Philippines, and Saudi Arabia [26]. Qualitative studies likewise highlighted educators’ preparedness and AI-related experience as important influences on their perceptions [30, 35]. However, experience was not universal; in a study of South Korean nurse educators, over one-third of the educators reported no experience of using GenAI despite generally expressing support for its use [34].

Greater knowledge and experience did not eliminate concerns about GenAI. Rather, educators with more experience often articulated more specific ethical, pedagogical, and professional concerns [33]. Reported challenges included the risk of inaccurate or unreliable information, reduced teacher–student interaction, difficulties maintaining academic integrity, and concerns regarding appropriate oversight of assessment processes [27, 33]. Educators also expressed concerns that students’ reliance on GenAI could weaken critical thinking skills, clinical reasoning, and the ability to prioritise patients’ needs in practice [29, 38]. Some educators worried that GenAI might diminish the traditional role of the nurse educator or potentially replace aspects of teaching practice [27, 34, 35]. Nevertheless, educators were generally supportive of students’ use of GenAI when it was applied appropriately and in ways that aligned with professional standards. To address these challenges, educators highlighted the need for tools to detect AI-generated content and support academic integrity [38]. Collaboration between nurse educators, clinical experts, and AI developers was also proposed as a means of strengthening AI preparedness and supporting responsible implementation of GenAI in nurse education [35]. Educators raised concerns regarding equity, pointing to unequal access to digital technologies and variations in AI literacy among both educators and students [38].

### Theme 2: Nurse educators’ competence and ways of using Generative AI

Reported competence in use of GenAI among nurse educators was linked to their experience, technical skills, and knowledge, as well as their position within the educational hierarchy [30]. Moreover, organizational policies on the use of AI were said to constitute a prerequisite for faculty competence, enabling them to be confident in applying GenAI in teaching.

Two included studies investigated the concept of competence by comparing it with constructs of innovation and creativity in the context of nurse education [28, 31]. Evidence of a positive predictive relationship between GenAI competence and pedagogical innovation, such as educators’ engagement in technology integration and use of new teaching methods was found [28]. A survey in Saudi Arabia found that nurse educators’ AI competence self-efficacy (confidence in using AI) was significantly associated with the evidence-based practice climate/knowledge [31]. A higher AI competence self-efficacy improved an educator’s ability to leverage EBP climate for more creative teaching.

The use of GenAI may be perceived as controversial owing to uncertainties about its accuracy [38]. However, exposure to and use of GenAI seem to play a central role in educators’ trust in technology and their self-efficacy in using it [26, 31].

Educators have underscored the importance of situating GenAI use within local practices, as well as the need for culturally appropriate adaptation to the contextual environment [31]. However, GenAI-use patterns among nursing educators were broadly consistent across regions—across the Middle Eastern and Asian samples (Saudi-Arabia, the Philippines, India, Egypt) as well as in the Jordan and U.S. samples -suggesting similar adoption practices [26, 27]. In the study by Saleh et al. [27] the use for teaching, personal learning, and research was distributed equally among the 391 participants across the USA and Jordan.

It was apparent that nursing educators used GenAI in a wide range of pedagogical activities. This included designing instructions and lessons [33, 35] simplifying research content [33], creating assignments, tests and examinations [32, 33], and supporting curriculum and syllabus development [30, 33]. They also employed GenAI for their own benefit, to enhance teaching practices and summarize documents and support academic writing [36], generate ideas and retrieve information and monitor students’ appropriate use of GenAI [35], and identify learning needs and evaluate student performance [34]. However, nurse educators called for increased knowledge resources and policies regarding the proper use of GenAI in nurse education to guide them [38].

## Discussion

This systematic review aimed to synthesize evidence on how nurse educators experience using GenAI in teaching. Our results indicate that nurse educators perceive GenAI as both an emerging opportunity and a source of concern owing to the uncertain accuracy of GenAI. Some included studies made a distinction between the awareness of limitations of GenAI and perceptions of its challenges and opportunities. Awareness is increased by use, whereas perceptions of challenges and opportunities were not necessarily grounded in extensive experience. The results show ambivalence towards the use of GenAI that relates to nurse educators’ experience, knowledge, competence, and preparedness. While GenAI is recognized for its potential to enhance efficiency and support pedagogical development, educators also highlighted ethical, legal and educational considerations related to academic integrity and the evolving role of nurse educators. These results may have implications for how nurse educators facilitate students’ development of critical clinical reasoning skills.

Nurse educators expressed a concern about losing the traditional teacher role to GenAI, transferring control of the teaching and learning process, and along with that, decreased teacher-student interactions. It implies a need for pedagogical development that is concurrent with technical development and for educators to re-think their teaching practice, for example by integrating learning activities that require students to critically evaluate and justify AI-generated responses [39, 40]. Although students and educators differ in their objectives and purposes for using GenAI, similarities in their attitudes towards its use has been found in earlier studies [14]. Both groups expressed a combination of curiosity and caution and shared concerns about ethical implications and academic integrity [14], which is consistent with the findings of the present review.

### Key finding 1: Educators’ experience with GenAI

The results indicate that nurse educators’ experience and actual use of GenAI are associated with a more balanced appraisal and greater trust of its usefulness. There was distrust of the accuracy of GenAI, with exploration of both opportunities and risks. This aligns with a descriptive study of participants’ development of trust [1]. These authors state that frequency of use, length of use, and self-rated proficiency are all significant predictors of trust.

This raises important questions regarding GenAI, how professional development and experiential learning might reduce unfounded concerns and yet support more informed pedagogical decision-making in the nurse educator field.

### Key finding 2: Tension between efficiency and core professional competence in nurse education

A key finding is the imbalance between nurse educators’ experiences and preparedness: the educators report limited generative AI competency while simultaneously expecting students to use these tools responsibly and professionally. This tension highlights the need for new institutional policies, faculty development, and pedagogical frameworks that clearly articulate the role of generative AI in the nurse education curriculum. We note that many tertiary institutions have guidelines only on how GenAI may or may not be used, although rarely describe how compliance should be monitored or how competence should be ensured among faculty and students [5]

GenAI is described as a powerful aide for saving time, structuring content and managing administrative tasks. At the same time, nurse educators voiced concerns that such technologies may undermine professional values such as critical thinking, clinical judgement and the human dimension of the caring relationship. This highlights a fundamental tension between technological efficiency and the value base of professional nursing, underscoring the need for pedagogical strategies that position GenAI as a tool for strengthening rather than displacing professional competence. In teaching, this can be described as didactic dissonance [41]. However, there are emerging frameworks that promote a shift and transition from basic GenAI literacy to a GenAI-augmented clinical practice [42]. Similarly to educators, Ng et al. [43] also demonstrated the importance of medical students maintaining their own judgement and evaluating GenAI’s output against knowledge, as well as assessing appropriateness within the given context.

### Key Finding 3: Organizational support, clear policy guidelines and equitable professional development necessary

The studies reveal substantial variation in GenAI competence linked to age, educational background, organizational role and access to policies or institutional guidance. The absence of a clear policy framework and training contributes to staff uncertainty regarding ethics, legal considerations and the assessment of students’ work. Further development is necessary as Generative AI is no longer a standalone tool but is increasingly embedded in widely used systems such as learning management systems (LMS) and online course platforms [44].

There is a need for HEIs to assume more responsibility for professional development, implement a structured institutional AI policy and enable equitable access to GenAI tools for both educators and students. This follows guidance by Erhardt et al. [5], who emphasize that while existing policies primarily address academic integrity, there remains a clear need to develop guidance related to pedagogical integration, governance and ethical use of generative AI in HEIs. Moreover, Castonquay [12] emphasizes the importance of collaborating with professional nursing associations to ensure that the core values and disciplinary identity of nursing remain visible, are protected and not marginalized within broader conversations about GenAI integration.

### Strengths and limitations

This review is situated within a pluralistic paradigm, drawing on constructivist, post-positivist, and pragmatic assumptions to guide the processes of evidence appraisal and synthesis in the context of nurse education [45]. All authors have professional backgrounds in nursing and extensive experience as nurse educators, providing contextual insight into the educational aspects represented in the screened and included articles [46]. Reflecting our search strategy, the included studies situated the scope of this review firmly within the academic nurse education rather than clinical teaching contexts. As such, the results may not be fully transferable to clinical nurse education environments, where teaching roles, responsibilities, and contextual factors differ.

All the included studies were appraised using validated quality assessment tools, including the JBI Critical Appraisal Checklists for qualitative (*n* = 5) and cross-sectional (quantitative) studies (*n *= 6), and the Mixed Methods Appraisal tool (MMAT) for mixed methods studies (*n* = 2) [19, 24]. Overall, the studies were judged to be of acceptable methodological quality, strengthening confidence in the credibility of the review results. Nevertheless, this review has several methodological limitations that should be considered. Although a comprehensive search strategy was employed, relevant studies may have been missed due to the rapidly evolving terminology used to describe GenAI, which may have limited retrieval. Furthermore, only studies published in the selected databases and meeting the predefined eligibility criteria were included. In alignment with Higgins et al., [16], decisions regarding study selection were undertaken independently by reviewers and resolved through discussion. Judgement was required, particularly when determining whether participants met the review definition of nurse educators working in HEI and whether studies aligned with the operational definition of GenAI adopted in the review. Moreover, the inclusion of English-language studies only may have resulted in underrepresentation of relevant evidence from other linguistic and cultural contexts. The included studies were heterogeneous in terms of design, educational context, and conceptualisation of GenAI, requiring interpretive decisions during the thematic synthesis. Although themes were continuously reviewed and refined through consensus among the review team to enhance reflexivity and analytical rigour, alternative interpretations of the data are possible [25].

Furthermore, as all included studies focused exclusively on GenAI, the results may not be transferable to educators’ experiences of other forms of AI used in education which also may present different educational opportunities and challenges. In the reviewed studies, nurse educators occupy diverse professional positions, ranging from teaching-focused to research-intensive and leadership positions, which may shape their exposure to, experiences, perceived relevance of and engagement with GenAI. Consequently, the results reflect a heterogenous and rapidly evolving landscape rather than a uniform experience. While this may limit the transferability of specific findings across contexts, it also strengthens the overall conclusion that GenAI use is becoming increasingly embedded within higher nurse education internationally.

## Conclusion

This review highlights that nurse educators perceive GenAI as both a promising and challenging resource in nurse education. While GenAI was considered to support teaching efficiency, academic work, and educational innovation, concerns remained regarding academic integrity, ethical responsibilities, critical thinking, and professional judgement. The findings further suggest that successful integration of GenAI depends not only on educators' perceptions but also on their competence, preparedness, and opportunities for meaningful engagement with technology. Experience, technical skills, institutional policies, and professional development appear to be important enablers of confident and responsible GenAI use. As GenAI continues to evolve within nurse education, future research should examine how educational institutions can support competence development and implementation strategies that maximise the benefits of GenAI while safeguarding core nurse educational values.

## Supplementary Information

Supplementary Material 1

Supplementary Material 2

## Data Availability

No datasets were generated or analysed during the current study.
