# Supplementary material for: Nurse educators' experiences and perceptions using generative artificial intelligence: a systematic review
Source: BMC Med Educ. 2026 Aug 15;26:1325. doi: 10.1186/s12909-026-10113-0 (PMC13479907; doi:10.1186/s12909-026-10113-0)
Supplement: Supplementary file 1 [file 12909_2026_10113_MOESM1_ESM.docx]

| **Database** | **Search date** | **Search string and filters** | **Number of hits** |
| --- | --- | --- | --- |
| **Cinahl** | 2025-03-12 | (((MH "Artificial Intelligence, Generative+")) OR ((TI generative artificial intelligence) OR (AB generative artificial intelligence)) OR ((TI generative AI) OR (AB generative AI)) OR ((TI genAI) OR (AB genAI)) OR ((TI gen AI) OR (AB gen AI)) OR ((TI AI-based) OR (AB AI-based)) OR ((TI chatbot*) OR (AB chatbot*)) OR ((TI chat bot*) OR (AB chat bot*)) OR ((TI machine intelligence) OR (AB machine intelligence)) OR ((TI machine learning) OR (AB machine learning)) OR ((TI Bing AI) OR (AB Bing AI)) OR ((TI ChatGPT) OR (AB ChatGPT)) OR ((TI Chat GPT) OR (AB Chat GPT)) OR ((TI Copilot) OR (AB Copilot)) OR ((TI Da Vinci) OR (AB Da Vinci)) OR ((TI DeepL) OR (AB DeepL)) OR ((TI DeepSeek) OR (AB DeepSeek)) OR ((TI Gemini) OR (AB Gemini)) OR ((TI Perplexity) OR (AB Perplexity)) OR ((TI Pi AI) OR (AB Pi AI)) OR ((TI Tactiq) OR (AB Tactiq))) AND (((MH "Education, Nursing+")) OR ((MH "Faculty, Nursing")) OR ((MH "Nurse Educators+")) OR ((TI nurs* educat*) OR (AB nurs* educat*)) OR ((TI nurs* facult*) OR (AB nurs* facult*)) OR ((TI nurs* lecturer*) OR (AB nurs* lecturer*)) OR ((TI nurs* mentor*) OR (AB nurs* mentor*)) OR ((TI nurs* pedagog*) OR (AB nurs* pedagog*)) OR ((TI nurs* supervis*) OR (AB nurs* supervis*)) OR ((TI nurs* teach*) OR (AB nurs* teach*)) OR ((TI nurs* tutor*) OR (AB nurs* tutor*)))  Filters: 2022-01-01-2025-03-12, English | 140 |
| **Eric** | 2025-10-08 | (((subject("nursing education") OR subject(nurs* educat*) OR noft(nurs* facult*) OR noft(nurs* lecturer*) OR noft(nurs* mentor*) OR noft(nurs* pedagog*) OR noft(nurs* supervis*) OR noft(nurs* teach*) OR noft(nurs* tutor*)) AND pd(20220101-20251008)) AND ((Subject("artificial intelligence") OR noft(artificial intelligence) OR noft(AI) OR noft(genAI) OR noft(AI-based) OR noft(chatbot*) OR noft(chat bot*) OR noft("machine intelligence") OR noft("machine learning") OR noft(Bing AI) OR noft(ChatGPT) OR noft(Chat GPT) OR noft(Copilot) OR noft("Da Vinci") OR noft(DeepL) OR noft(DeepSeek) OR noft(Gemini) OR noft(Perplexity) OR noft(Pi AI) OR noft(Tactiq)) AND (la.exact("ENG") AND pd(20220101-20251008)))) AND stype.exact("Scholarly Journals")  Filters: 20220101-20251008, English | 20 |
| **PubMed** | 2025-03-06 | ((((((((((((((((((((("artificial intelligence"[MeSH Terms]) OR (generative artificial intelligence[Title/Abstract])) OR (generative AI[Title/Abstract])) OR (genAI[Title/Abstract])) OR (gen AI[Title/Abstract])) OR (AI-based[Title/Abstract])) OR (chatbot*[Title/Abstract])) OR (chat bot*[Title/Abstract])) OR (machine intelligence[Title/Abstract])) OR (machine learning[Title/Abstract])) OR (Bing AI[Title/Abstract])) OR (ChatGPT[Title/Abstract])) OR (Chat GPT[Title/Abstract])) OR (Copilot[Title/Abstract])) OR (Da Vinci[Title/Abstract])) OR (DeepL[Title/Abstract])) OR (DeepSeek[Title/Abstract])) OR (Gemini[Title/Abstract])) OR (Perplexity[Title/Abstract])) OR (Pi AI[Title/Abstract])) OR (Tactiq[Title/Abstract])) AND (((((((((("education, nursing"[MeSH Terms]) OR ("faculty, nursing"[MeSH Terms])) OR (nurs* educat*[Title/Abstract])) OR (nurs* facult*[Title/Abstract])) OR (nurs* lecturer*[Title/Abstract])) OR (nurs* mentor*[Title/Abstract])) OR (nurs* pedagog*[Title/Abstract])) OR (nurs* supervis*[Title/Abstract])) OR (nurs* teach*[Title/Abstract])) OR (nurs* tutor*[Title/Abstract]))  Filters: from 2022-2025, English | 189 |
| **Web of Science** | 2025-03-17 | (TS=("generative artificial intelligence" OR AI OR genAI OR "gen AI" OR "AI-based" OR chatbot* OR "chat bot*" OR "machine intelligence" OR "machine learning" OR "Bing AI" OR ChatGPT OR "Chat GPT" OR Copilot OR "Da Vinci" OR DeepL OR DeepSeek OR Gemini OR Perplexity OR " Pi AI" OR Tactiq) AND TS=(“nurs* educat*” OR “nurs* facult*” OR “nurs* lecturer*” OR “nurs* mentor*” OR “nurs* pedagog*” OR “nurs* supervis*” OR “nurs* teach*” OR “nurs* tutor*”))  Filters: 2022 or 2023 or 2024 or 2025, English | 253 |
