# Supplementary material for: Nurse educators' experiences and perceptions using generative artificial intelligence: a systematic review
Source: BMC Med Educ. 2026 Aug 15;26:1325. doi: 10.1186/s12909-026-10113-0 (PMC13479907; doi:10.1186/s12909-026-10113-0)
Supplement: Supplementary file 2 [file 12909_2026_10113_MOESM2_ESM.docx]

**Table X. Methodological quality appraisal of the included studies**

**Qualitative studies, JBI Critical Appraisal Checklist, (Porrit et al., 2024)**

| **Quality criteria** | **Durmus et al.** | **Kim et al.** | **Lane et al.** | **Rony et al.** | **Summers et al.** |  |
| --- | --- | --- | --- | --- | --- | --- |
| Congruity between philosophical perspective and methodology | Yes | Yes | Unclear | Yes | Yes |  |
| Congruity between methodology and research question or objectives | Yes | Yes | Yes | Yes | Yes |  |
| Congruity between methodology and the data collection methods | Yes | Yes | Yes | Yes | Yes |  |
| Congruity between methodology and data analysis | Yes | Yes | Yes | Yes | Yes |  |
| Congruity between methodology and the interpretation of results | Yes | Yes | Yes | Yes | Yes |  |
| Statement locating researcher positioning culturally or theoretically | Yes | Yes | Yes | Yes | Yes |  |
| Influence of the researcher on the research, and vice versa addressed | Yes | Yes | Yes | Yes | Unclear |  |
| Participants and their voices adequately represented | Yes | Yes | Yes | Yes | Yes |  |
| Ethical approval by an appropriate body or according to current criteria reported | Yes | Yes | Yes | Yes | Yes |  |
| Conclusions drawn in the research report flow from the analysis, or interpretation, of the data | Yes | Yes | Yes | Yes | Yes |  |
| **Overall quality rating** | **High** | **High** | **Moderate** | **High** | **Moderate** |  |

**Included Cross- sectional studies, JBI Critical Appraisal Checklist, (Barker et al., 2024)**

| **Quality criteria** | **Almaki et al.** | **Asal et al.** | **Ehmke et al.** | **El-Sayed et al.** | **Hong et al.** | **Saleh et al.** |
| --- | --- | --- | --- | --- | --- | --- |
| Clearly defined inclusion criteria | Yes | Yes | Yes | Yes | Yes | Yes |
| Study subjects and settings described | Yes | Yes | Yes | Yes | Yes | Yes |
| Exposure measured in a valid and reliable way | Yes | Yes | Yes | Yes | Yes | Yes |
| Objective, standard criteria for measurements used | Yes | Yes | No | Yes | Yes | Yes |
| Identification of confounding factors | Yes | Yes | Yes | Yes | Yes | Yes |
| Strategies to deal with confounding factors stated | Yes | Yes | Yes | Yes | No | Yes |
| Outcome measured in a valid and reliable way | Yes | Yes | Yes | Yes | Yes | Yes |
| Appropriate statistical analysis | Yes | Yes | Yes | Yes | Yes | Yes |
| **Overall quality rating** | **High** | **High** | **Moderate** | **High** | **Moderate** | **High** |

**Included Mixed-methods studies, MMAT, 2018, (Hong et al., 2018)**

| **Quality criteria** | **Eltaybani et al.** | **Hashish et al.** |
| --- | --- | --- |
| Clear research questions | Yes | Yes |
| The collected data address the research questions? | Yes | Yes |
| Adequate rationale for mixed-methods design | Yes | Yes |
| Integration of different components | Yes | Yes |
| Interpretation of the outputs of the integration of different components | Yes | Yes |
| Divergences and inconsistencies addressed | Yes | Yes |
| Adherence to quality criteria of each method | Yes | Yes |
| **Overall quality rating** | **High** | **High** |
